# The impact of the COVID-19 vaccination programme on symptomatic and severe SARS-CoV-2 infection during a period of Omicron variant dominance in Ireland, December 2021 to March 2023

**DOI:** 10.2807/1560-7917.ES.2024.29.28.2300697

**Published:** 2024-07-11

**Authors:** Louise Marron, Alberto Mateo-Urdiales, Joan O’Donnell, Eve Robinson, Lisa Domegan

**Affiliations:** 1 Health Service Executive-Health Protection Surveillance Centre, Dublin, Ireland; 2 European Centre for Disease Prevention and Control (ECDC) Fellowship Programme, Field Epidemiology path (EPIET), ECDC, Stockholm, Sweden; 3 Department of Infectious Diseases, Istituto Superiore di Sanità (Italian National Institute of Health), Rome, Italy

**Keywords:** COVID-19, averted outcomes, vaccination coverage, vaccination programmes, winter preparedness

## Abstract

**Background:**

As Ireland prepared for an autumn 2023 COVID-19 vaccination booster campaign, there was concern that vaccine fatigue would affect uptake, which has been abating.

**Aim:**

This study aimed to quantify the direct impact of the COVID-19 vaccination programme in Ireland on averted COVID-19-related outcomes including symptomatic presentations to primary care/community testing centres, emergency department (ED) presentations, hospitalisations, intensive care unit (ICU) admissions and deaths, in individuals aged ≥ 50 years, during Omicron dominance.

**Methods:**

We conducted a retrospective observational COVID-19 vaccine impact study in December 2021–March 2023 in Ireland. We used national data on notified outcomes and vaccine coverage, as well as vaccine effectiveness (VE) estimates, sourced from the World Health Organization’s live systematic review of VE, to estimate the count and prevented fraction of outcomes in ≥ 50-year-olds averted by the COVID-19 vaccination programme in this age group.

**Results:**

The COVID-19 vaccination programme averted 48,551 symptomatic COVID-19 presentations to primary care/community testing centres (36% of cases expected in the absence of vaccination), 9,517 ED presentations (53% of expected), 102,160 hospitalisations (81% of expected), 3,303 ICU admissions (89% of expected) and 15,985 deaths (87% of expected).

**Conclusions:**

When Omicron predominated, the COVID-19 vaccination programme averted symptomatic and severe COVID-19 cases, including deaths due to COVID-19. In line with other international vaccine impact studies, these findings emphasise the benefits of COVID-19 vaccination for population health and the healthcare system and are relevant for informing COVID-19 booster vaccination programmes, pandemic preparedness and communicating the reason for and importance of COVID-19 vaccination in Ireland and internationally.

**Key public health message**

**What did you want to address in this study and why?**

In this study, we wanted to assess the impact that the COVID-19 vaccination programme has had in Ireland during the Omicron period in people aged ≥ 50 years. We estimated the number of symptomatic and severe cases of COVID-19 that have been prevented by vaccination to inform future vaccination recommendations, as well as communication with healthcare professionals and the public about the reason for additional COVID-19 boosters.

**What have we learnt from this study?**

The COVID-19 vaccination programme in Ireland prevented illness and death due to COVID-19 in people aged ≥ 50 years. COVID-19 vaccination therefore protected population health and the healthcare system, underscoring through impact estimates the importance of vaccination.

**What are the implications of your findings for public health?**

The uptake of additional COVID-19 vaccination booster doses has been lower compared with the uptake of the primary vaccination course and first boosters. The findings of this study can be used to inform future COVID-19 vaccination policies, planning for winter seasons, pandemic preparedness, and communication about the importance of ongoing COVID-19 vaccination programmes in Ireland and in other countries.

## Introduction

The COVID-19 pandemic presented an unprecedented challenge to populations, governments and health and social care systems in Ireland and worldwide [1]. Nationally and internationally, there has been a reduction in severe outcomes following COVID-19 infection, largely attributed to the roll-out of effective COVID-19 vaccines [2]. In Ireland, since December 2021, the severe acute respiratory syndrome coronavirus 2 (SARS-CoV-2) Omicron variant and various sub-lineages and recombinants, have predominated [3]. Globally, reduced severity of SARS-CoV-2 infection has been reported for Omicron compared with other variants (e.g. Delta), likely due to increased population-level immunity, protection from vaccination and prior infection [4]. However, enhanced infectivity, immune evasion, and reduced vaccine effectiveness (VE) against Omicron variants have also been reported [5].

The COVID-19 vaccination programme commenced in Ireland in December 2020. Based on recommendations from the National Immunisation Advisory Committee (NIAC), vaccination roll-out was prioritised to population groups with a higher risk of complications from or exposure to SARS-CoV-2 infection [6]. As in other European countries, such groups included individuals in residential care facilities, healthcare workers (HCWs), those with immunocompromise and underlying medical conditions and those in older age groups, beginning with people aged ≥ 85 years before progressing in a stepwise approach to those in younger age cohorts [7,8]. Further detail on the COVID-19 vaccination programme in Ireland is included in the Supplementary Materials’ section entitled ‘The roll-out of the COVID-19 vaccination programme in Ireland’.

By winter 2021/22, when the first Omicron variant (BA.1) emerged as the dominant circulating variant, 94% of the population in Ireland aged ≥ 18 years were fully vaccinated with a primary COVID-19 vaccine course [9], and the first booster campaign was being rolled out by priority group, according to NIAC recommendations [6].

During 2022, the COVID-19 vaccination booster campaign continued with the recommendation for second and third booster doses for those in priority groups according to NIAC guidance [10]. In Ireland, mRNA, viral vector and protein subunit vaccines have been administered, however, mRNA vaccines have been preferentially used for booster vaccination [6]. Prior to September 2022, only monovalent mRNA vaccines were available, however from September 2022, bivalent mRNA vaccines were preferentially offered when available [6].

In the current post-acute phase of the COVID-19 pandemic, vaccination strategies continue to evolve [11]. In Ireland, and internationally, vaccination recommendations to target certain population groups have continued to be informed by the risk of severe illness in these groups e.g. those in older age groups, and the effectiveness of the vaccine at reducing severe outcomes [12-14]. In Ireland in autumn 2023, a COVID-19 booster vaccine was recommended for all ≥ 50-year-olds, HCWs and those aged ≥ 5 years with immunocompromise or a high risk of serious illness from SARS-CoV-2 infection [6,15].

As Ireland prepared for the autumn booster campaign, there was concern that vaccine fatigue would impact vaccine coverage, which has been abating [16,17]. As COVID-19 vaccination was offered to all ≥ 50-year-olds, understanding and quantifying the impact of COVID-19 vaccination in this key target group was important to inform future vaccination strategies in Ireland and communication materials for healthcare professionals and the public, including clear communication about the rationale for recommendations for additional booster doses for the 2023/24 booster campaign and for future autumn/winter COVID-19 vaccination programmes.

The aim of this study was to quantify the direct impact of the COVID-19 vaccination programme in people aged ≥ 50 years in Ireland on COVID-19 cases presenting to primary care and community testing centres with symptoms, emergency department (ED) presentations and hospitalisations with COVID-19 and intensive care unit (ICU) admissions and deaths due to COVID-19 in this age group, during a period of Omicron variant dominance from December 2021 until March 2023.

## Methods

### Study design

We conducted a retrospective observational COVID-19 vaccine impact study, estimating, for those aged ≥ 50 years, the number of events averted by vaccination in this age group and the disease prevented fraction (PF). We estimated the number of averted events (NAE) using a method originally used to assess the impact of influenza vaccination in Portugal [18], which has been adapted and used internationally to estimate the impact of COVID-19 vaccination [19,20]. The study was during a period of Omicron dominance from week 51 2021 (week beginning 20/12/2021) when Omicron became the dominant circulating variant in Ireland [3], to week 12 2023 (week ending 26/03/2023), which was immediately before a change in national COVID-19 testing policies [21]. An overview of COVID-19 testing recommendations and non-pharmaceutical interventions (NPI) recommended between December 2021 and April 2023 is provided in the Supplementary Materials’ section entitled ‘Overview of non-pharmaceutical interventions during Omicron dominance in Ireland: December 2021 to 1st April 2023’.

### Data sources and data extraction

Data on key outcomes, VE and COVID-19 vaccine coverage were required.

#### Outcome data

Notified symptomatic COVID-19 cases who consulted primary care or attended a COVID-19 community testing centre, as well as ED presentations and hospitalisations with COVID-19 and ICU admissions and deaths due to COVID-19 reported in those aged ≥ 50 years were extracted from Ireland’s Computerised Infectious Disease Reporting system (CIDR). CIDR is the national, validated surveillance repository for notifiable diseases data in Ireland. Only laboratory-confirmed cases were included. A laboratory-confirmed case was a case for whom SARS-CoV-2 virus genetic material was detected by PCR. Anonymised case-based data were used to calculate weekly counts of each outcome and a weekly rolling average number of outcomes observed over 3 weeks.

Within CIDR, a *patient type* variable was used to classify COVID-19 cases according to whether they attended a general practitioner (GP)/community testing centre or an ED or were hospitalised. These outcome categories were exclusive. Death and ICU admission status were recorded using different variables: outcome and ICU admission. Cases who were subsequently admitted to ICU or who died were excluded from the symptomatic presentation to GP/community testing centres, ED presentation and hospitalisation outcomes to more accurately estimate the impact on each outcome exclusively. Cases who were admitted to ICU and who subsequently died were included in the ICU admission outcome.

Outcome 1, symptomatic COVID-19 cases presenting to primary care/community testing centres, was defined as a notified laboratory-confirmed COVID-19 case who was symptomatic, and either consulted their GP or attended a community COVID-19 testing centre. Those whose symptom status was unknown were excluded from this outcome. The symptomatic variable was used only for the symptomatic presentation to primary care/community testing centres outcome category, to specifically exclude those who were asymptomatic who had been referred for testing in community testing centres due to being a close contact of a COVID-19 case. Due to suboptimal completeness of the symptomatic variable within the national surveillance database, the symptomatic variable was not used for the classification of outcomes 2–5.

Outcome 2, ED presentation was defined as a notified laboratory-confirmed COVID-19 case who presented for emergency care in an ED due to or with COVID-19.

Outcome 3, hospitalisation, was defined as a notified laboratory-confirmed COVID-19 case who was hospitalised due to or with COVID-19.

Within ED presentation (outcome 2) and hospitalisation (outcome 3) variables, it was not specified within the variables whether the case presented to ED or was hospitalised due to COVID-19 or due to another cause and had COVID-19 detected on ED presentation or on hospitalisation.

Outcome 4, ICU admission, was defined as a notified laboratory-confirmed COVID-19 case with an ICU admission due to COVID-19. In Ireland, a universal surveillance system is in place for monitoring all COVID-19 cases admitted to ICU due to COVID-19. To be classified as an ICU admission due to COVID-19, COVID-19 infection must be stated as the primary source of the ICU admission by the ICU medical team.

Outcome 5, death, was defined as a death where COVID-19 was a direct or contributing cause of death resulting from a clinically compatible illness in a confirmed COVID-19 case, unless there was a clear alternative cause of death that could not be related to COVID-19. Deaths where there was a clear alternative cause of death (e.g. a death due to road traffic accident) are not notified as COVID-19 deaths nationally and were therefore excluded from these analyses. In a sensitivity analysis, the definition of a COVID-19 death was changed to include those classified only as having a cause of death field completed within CIDR where death was specified as being due to COVID-19 to compare results between this definition and the study outcome.

#### Vaccine effectiveness data

The International Vaccine Access Center (IVAC), John Hopkins Bloomberg School of Public Health and the World Health Organization (WHO) are conducting an ongoing, live systematic review of COVID-19 VE studies [22]. The literature search strategy and inclusion criteria for this systematic review and live VE estimates are available from the IVAC VIEW-hub platform (https://view-hub.org/resources) [23]. VE estimates (reported up to 21 July 2023) were extracted from this platform. The methodology was informed by the WHO methodology for extracting VE estimates [22], by other studies that have used IVAC data [24], and by consultation with colleagues working on vaccine impact studies internationally [19,20]. Study specific inclusion criteria for the selection of studies for inclusion and criteria for selection of VE estimates are included in the Supplementary Materials’ section entitled ‘Study specific inclusion criteria’.

VE estimates were extracted for each outcome by vaccination status (primary course, first booster, second booster or third booster). Given that the coverage of the primary course and first booster was high at the beginning of the study period, as illustrated in Figure S1 in the Supplementary Materials, the latest reported VE estimates for the primary course and booster 1 (by time since vaccination) were used. Extracted VE estimates were collated, and an average of the VE point estimates against each study outcome for each vaccination status was calculated. To account for uncertainty around VE estimates, a minimum and maximum VE estimate was calculated around the average VE point estimate by varying the VE by +/− 10%. In this sensitivity analysis, where VE was already over 90%, the maximum VE was estimated at 99.9%. An additional sensitivity analysis was conducted by extracting the 95% confidence intervals (CIs) reported around each point estimate included and calculating an average upper and lower CI around each point estimate of VE.

#### COVID-19 vaccination coverage data

COVID-19 vaccination coverage data for Ireland were sourced from the Health Protection Surveillance Centre (HPSC) using data reported to the European Surveillance System (TESSy) and the European surveillance portal for infectious diseases (EpiPulse), coordinated by the European Centre for Disease Prevention and Control (ECDC). The HPSC reports data on COVID-19 vaccine doses administered in Ireland to TESSy/EpiPulse each week. The TESSy/EpiPulse data contain weekly numbers of vaccinations by age group and the dose of vaccine administered, they do not contain information on time since vaccination or sex. Weekly cumulative population vaccination counts and coverage in those aged ≥ 50 years by vaccination dose (complete primary course, first booster, second booster, third booster, fourth booster) were calculated. As the cumulative coverage of the fourth booster at the end of the study period (week 12 2023) was 0.1% in those aged ≥ 50 years, this study was restricted to assessing the impact of the complete primary vaccination course, first booster, second booster and third booster. Cumulative weekly coverage for each exclusive vaccination status (e.g. primary course only, primary course and first booster only, primary course and first and second booster only, primary course and first, second and third booster only) was calculated.

The definitions of vaccination status in this study aligned with national definitions which are described in the Supplementary Material’s subsection entitled ‘Definition of vaccination status used for COVID-19 vaccination coverage calculations’ [16]. For calculation of cumulative weekly vaccination coverage, the most up to date, Central Statistics Office (CSO) census single year estimates available in Ireland (updated in May 2023) were used as the population denominator [25]. In circumstances where, due to data quality issues, the cumulative weekly count exceeded the population denominator, the count was capped at the population denominator to ensure the cumulative vaccination coverage did not exceed 100%.

### Data analysis

The impact of COVID-19 vaccination on each outcome was estimated using the formula below adapted from other vaccine impact studies [18-20]. The NAEs for each vaccination status (k), the three weekly rolling average of observed events/outcomes (*n*), vaccine coverage for a specific vaccination status (*VC*) and the absolute VE against the event/outcome for a specific vaccination status were included in this formula. The weekly and total NAEs were calculated for the study period for each outcome by vaccination status (k); primary course (*pc*), first booster (*b1*), second booster (*b2*) and third booster (*b3*).

${NAE}_{k}=n\times \frac{VCVEk}{1-\left(VCVEpc\;+\;VCVEb1+VCVEb2+VCVEb3\right)}$

The total number of expected events in the absence of vaccination during the study period was calculated by summing the events observed and the events averted. The weekly and total age-specific cumulative incidence of observed and expected events per 100,000 population was calculated. The PF for each outcome (by vaccination status) was estimated using the formula:

$PF=\frac{NAE}{n+NAE}$

Minimum and maximum values for averted and expected counts, rates and PF for each outcome were calculated by varying the VE estimates by +/− 10%. These maximum and minimum values were reported as a range around each vaccine impact estimate. A sensitivity analysis using average 95% CIs around the point VE estimate was also undertaken. All data analyses were conducted in R version 4.2.1 [26].

## Results

### Vaccine coverage

**Figure 1:**
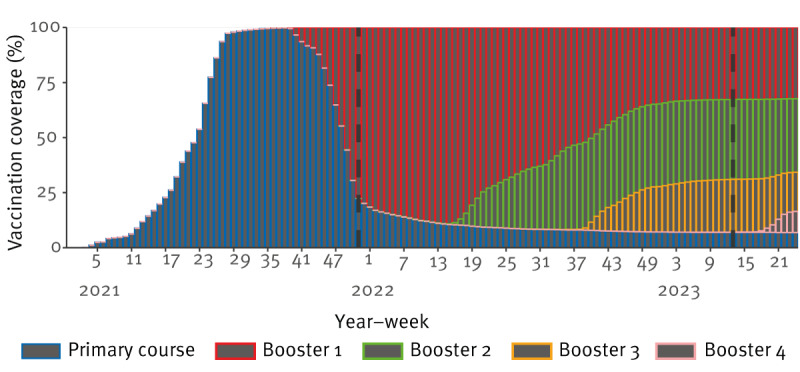
Weekly vaccination coverage by vaccination status in people aged ≥ 50 years, Ireland, week 53 2020–week 24 2023

At the beginning of the study period (week 51 2021), cumulative coverage of the primary COVID-19 vaccine course and booster 1 vaccines in those aged ≥ 50 years was 99.9% and 78.0% respectively. At the end of the study period (week 12 2023), cumulative coverage of booster 1 was 93.0%, booster 2 was 60.3% and booster 3 was 24.0%. During the study period those vaccinated with booster 1 following completion of the primary vaccination course contributed to the highest proportion of population COVID-19 vaccination coverage (Figure 1).

Dashed lines indicate the beginning and end of the study period.

### Vaccine effectiveness

Average VE point estimates for the Omicron variant calculated against symptomatic infection/cases [27-29], ED presentation [30-38], hospitalisation [28,36,39-46], ICU admission [41,43,47,48], and death [42-44,46-50] are shown in Table 1. Uncertainty estimates of +/− 10% were included around each average point estimate shown in Table 1. Table S7 in the Supplementary Materials’ section entitled ‘Sensitivity Analyses’ shows the average 95% CIs around each estimate used for a sensitivity analysis.

**Table 1:** Average COVID-19 vaccine effectiveness point estimates by specific vaccination status and outcome

Vaccine effectiveness (%) by outcome	Primary course^a^	Booster 1^a^	Booster 2^a^	Booster 3^a^
Symptomatic infection/cases^b^	20	40	22	26
Emergency department presentation	29	56	57	62
Hospitalisation	62	86	73	73
Intensive care unit admission	76	90	94	94
Death	72	90	84	84

Source: https://view-hub.org/resources [23].

^a ^Uncertainty estimates of +/− 10% included around all vaccine effectiveness estimates.

**^b^** Symptomatic infection/cases were not hospitalised and were not classified as having severe infection.

### Observed, averted and expected outcomes

**Figure 2:**
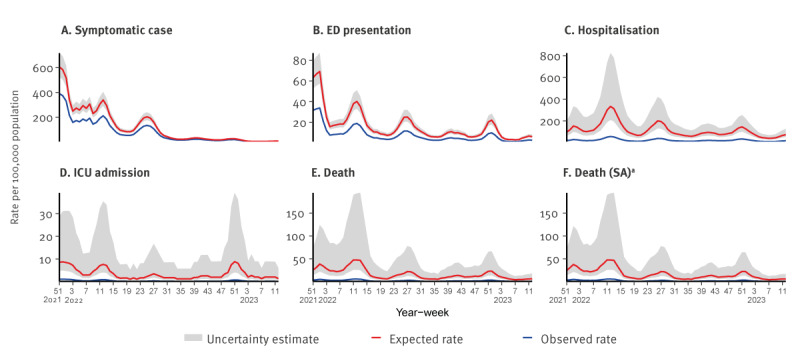
Observed and expected rate of outcomes per 100,000 population aged ≥ 50 years, including (A) symptoms, (B) ED presentation, (C) hospitalisation, (D) ICU admission and (E–F) death, Ireland, week 51 2021–week 12 2023

ED: emergency department; ICU: intensive care unit; SA: sensitivity analysis.

^a^ SA varying the definition of a death due to COVID-19 from death where COVID-19 was a direct or contributing cause of death (panel E) to deaths where it was specified in a cause of death variable that death was due to COVID-19 (panel F).

The observed and expected cumulative weekly incidence rate per 100,000 population aged ≥ 50 years with uncertainty estimates presented as a range for each outcome (including the sensitivity analysis varying the definition of a COVID-19 death) is shown in Figure 2.

During the study period (week 51 2021 to week 12 2023), the COVID-19 vaccination programme in Ireland averted an estimated 48,551 (30,296–73,574) symptomatic presentations to primary care/community testing centres; 9,517 (6,364–14,382) ED presentations; 102,160 (58,753–246,021), hospitalisations; 3,303 (1,500–15,801) ICU admissions and 15,985 (8,031–61,814) deaths (Table 2).

**Table 2:** Observed, averted and expected outcomes, as well as disease prevented fraction in those aged ≥ 50 years, Ireland, week 51 2021–week 12 2023

Outcome^a^	Observed	Averted	Averted range^b^	Expected	Expected range^b^	Observed rate^c^	Expected rate^c^	Expected rate^c^ range^b^	PF	PF range^b^
Symptomatic case^d^	86,570	48,551	30,296–73,574	135,121	116,866–160,144	5,103.9	7,966.3	6,890.0–9,441.6	0.36	0.26–0.46
ED presentation^e^	8,422	9,517	6,364–14,382	17,939	14,786–22,804	496.5	1,057.6	871.8–1,344.5	0.53	0.43–0.63
Hospitalisation	24,578	102,160	58,753–246,021	126,738	83,331–270,599	1,449.0	7,472.1	4,912.9–15,953.7	0.81	0.71–0.91
ICU admission	392	3,303	1,500–15,801	3,695	1,89–16,193	23.1	217.8	111.5–954.7	0.89	0.79–0.98
Death	2,429	15,985	8,031–61,814	18,414	10,460–64,243	143.2	1,085.6	616.7–3,787.6	0.87	0.77–0.96

ED: emergency department; ICU: intensive care unit; PF: disease prevented fraction; VE: vaccine effectiveness.

^a^ 3-week rolling average.

^b^ Range calculated by varying average VE point estimates by +/− 10%.

^c^ Per 100,000 population.

^d^ Symptomatic COVID-19 case presenting to primary care or community testing centre.

^e^ ED presentation excluded cases subsequently hospitalised or who were admitted to an ICU or who died.

The PF for each outcome (Table 2) estimated that the COVID-19 vaccination programme in Ireland prevented 36% (26–46%) of expected symptomatic COVID-19 presentations to primary care/community testing centres, 53% (43–63%) of expected ED presentations, 81% (71–91%) of expected hospitalisations and 89% (79–98%) of expected ICU admissions and 87% (77–96%) of expected deaths.

Analysis by vaccination status estimated that most averted outcomes occurred among those who had completed a primary course and the first booster (Table 3). Roll-out of second and third boosters began during the study period (Figure 1). Roll-out of the third booster began in week 38 2022 and during the study period, the third booster averted an estimated 260 (140–420) symptomatic presentations to primary care/community testing centres, 394 (269–590) ED presentations, 4,414 (2,660–8,839) hospitalisations, 228 (95–1,066) ICU admissions and 688 (361–2,227) deaths (Table 3).

**Table 3:** Averted outcomes in those aged ≥ 50 years by vaccination status, Ireland, week 51 2021–week 12 2023

Outcome^a^	Observed	Averted	Averted range^b^	PC^c^	PC range^b,c^	B1^d^	B1^d^ range^b^	B2^e^	B2^e ^range^b^	B3^f^	B3^f^ range^b^
Symptomatic case^g^	86,570	48,551	30,296–73,574	3,649	1,577–6,492	42,554	140–420	2,087	991–3,567	260	140–420
ED presentation^h^	8,422	9,517	6,364–14,382	634	343–1,081	7,098	269–590	1,391	939–2,100	394	269–590
Hospitalisation	24,578	102,160	58,753–246,021	8,117	4,439–20,604	73,362	2,660–8,839	16,268	9,570–35,008	4,414	2,660–8,839
ICU admission	392	3,303	1,500–15,801	314	143–1,510	2,150	95–1,066	611	260–2,921	228	95–1,066
Death	2,429	15,985	8,031–61,814	1,484	723–5,941	11,486	361–2,227	2,328	1,199–8,130	688	361–2,227

ED: emergency department; ICU: intensive care unit; VE: vaccine effectiveness.

^a^ 3-week rolling average.

^b^ Range calculated by varying average VE point estimates by +/− 10%.

^c^ PC: Outcome averted among people who had primary vaccination course only, with range.

^d^ B1: Outcome averted among people who had primary vaccination course and booster 1 only, with range.

^e^ B2: Outcome averted among people who had primary vaccination course, booster 1 and booster 2 only, with range.

^f^ B3: Outcome averted among people who had primary vaccination course, booster 1, booster 2 and booster 3 only, with range.

^g^ Symptomatic COVID-19 case presenting to primary care or community testing centre.

^h^ ED presentation excluded cases subsequently hospitalised or who were admitted to an ICU or who died.

Changing the outcomes to counts rather than 3-week rolling averages and varying the definition of a death due to COVID-19 did not impact the PF as illustrated in Tables S4, S5 and S6 of the Supplementary Materials’ section ‘Sensitivity Analyses’. A sensitivity analysis using average 95% CIs around the average VE point estimates produced a minimal change in the ranges reported around each vaccine impact estimate for each outcome as presented in Table S8 and S9 of the Supplementary Materials’ section ‘Sensitivity Analyses’.

## Discussion

This study used national surveillance data, national vaccine coverage data and average VE point estimates sourced from original published literature included in the WHO’s ongoing live systematic review of VE, to investigate COVID-19 vaccine impact [22]. The study estimated that during an Omicron period in Ireland, among those aged ≥ 50 years, the COVID-19 vaccination programme reduced symptomatic presentations to primary care/community testing centres by 36%, ED presentations by 53%, hospitalisations by 81%, ICU admissions by 89% and deaths by 87%.

Vaccine fatigue has been recognised as an emerging and important public health issue internationally, particularly as waning immunity and the emergence of new variants mean that booster doses are likely to continue to be required [51]. The findings of this study are therefore timely and relevant nationally and internationally for communication with the public and healthcare professionals about the importance of vaccination and the benefits of ongoing booster campaigns for recommended groups and may be useful for communication in future (autumn/winter) COVID-19 vaccination campaigns, to counter vaccine fatigue.

During the study period, completion of a primary course and booster 1 vaccination continued to avert symptomatic and severe cases of COVID-19 in the population aged ≥ 50 years. This finding emphasises the importance of the initial high national vaccine coverage with effective COVID-19 vaccines following the roll-out of the vaccination programme starting in December 2020 and is an important finding for public health communication about the rationale for and benefits of vaccination and for future pandemic preparedness and planning.

However, the impact of booster 1 vaccines reported in this study reflects the high proportion of the population vaccinated with booster 1 following completion of a primary vaccination course during this study period in Ireland. Importantly, this study also found that the ongoing COVID-19 vaccination campaign and subsequent booster doses (booster 2 and 3) had benefits at a population level particularly against serious outcomes (hospitalisation, ICU admissions and death). This finding, in conjunction with known waning of COVID-19 vaccine immunity [52], emphasises the importance of ongoing and timely COVID-19 booster vaccinations.

Nationally and internationally, the COVID-19 pandemic continues to impact population health and health services [1,53,54]. The effect of COVID-19 on health and care services is both direct and indirect as COVID-19 impacts healthcare capacity to provide both scheduled and unscheduled care [1,21,55]. This study examined a more recent period in the pandemic and demonstrated the value and ongoing benefits of the national COVID-19 vaccination programme in terms of preventing serious outcomes including hospitalisations and ICU admissions. If even the lower estimates of expected outcomes in the absence of vaccination had occurred during this study period, acute hospital and ICU capacity in Ireland would have been overwhelmed [56]. This likely would have resulted in morbidity and mortality in the population over and above what is reported in this study due to both COVID-19 and other illness. This finding is important for communication about the benefits of COVID-19 booster vaccination programmes to preserve and protect healthcare capacity, particularly during autumn/winter surge periods of respiratory infections.

The findings of this study are consistent with other published vaccine impact studies using similar and different methodologies [19,20,57]. Further assessment of vaccine impact is required both nationally and internationally and population-level vaccine impact studies should be conducted on an ongoing basis, both for COVID-19 and other vaccine preventable diseases e.g. influenza to assess the impact of vaccination programmes. These vaccine impact studies should, where possible, follow internationally agreed standardised methodologies and protocols to facilitate interpretation of results and comparison between countries. In Ireland, the HPSC is a partner in the ECDC’s Vaccine Effectiveness, Burden and Impact Studies of COVID-19 and Influenza (VEBIS) network and a member of the VEBIS consortium; this platform could be used to progress this work. Robust and reliable VE data are essential for undertaking vaccine impact studies and the certainty of VE estimates is critical to an accurate assessment of vaccine impact. National VE estimates would be a key enabler of undertaking vaccine impact studies and would improve the reliability and the generalisability of the findings to the population in Ireland.

Estimating COVID-19 vaccine impact is complex and questions remain, particularly about waning immunity by time since vaccination, contribution of immunity following prior infection, and the optimal timing of booster doses. However, the benefits of vaccination at a population level have been demonstrated in this vaccine impact study and the findings are relevant in Ireland and in Europe for public health communication, vaccination policy, planning for future (autumn/winter) COVID-19 vaccination programmes and for cost effectiveness analyses.

This study has nevertheless some limitations. It estimated the direct effect of the COVID-19 vaccination programme in those aged ≥ 50 years only. The benefits of vaccination in this age group and in younger age groups on transmission and on the preservation of healthcare capacity were not considered and therefore the benefits of the COVID-19 vaccination programme may have been underestimated. During the study period, uptake of COVID-19 booster vaccines was lower in younger age groups in Ireland, 34.5% in those aged ≥ 18 years as shown in Table S1 of the Supplementary Materials [16]. Changing vaccination coverage within different age groups in the population may have influenced the incidence of observed outcomes in those aged ≥ 50 years. Additionally, immunity from prior infection and hybrid immunity within the total population may have influenced the incidence of outcomes and the effectiveness of vaccinations in those aged ≥ 50 years.

Only PCR confirmed cases notified to the national surveillance database (CIDR) were included in this study. These data represent only those who sought care and were tested, this may have underestimated the counts of each outcome in the population and the impact of vaccination. For the outcomes of ED presentation and hospitalisation, confirmed cases may have had incidental SARS-CoV-2 infection which was not the cause of their experiencing the outcome, this may have overestimated the impact of the vaccination programme. Additionally, during the study period there were changes to testing, surveillance policies and national advice regarding NPIs, which may have impacted the number of notifications and the epidemiology of notified cases. To take this into account, the study period was restricted to a period of least change in testing, surveillance policies and national advice on NPIs.

There were challenges in the interpretation of VE estimates from primary studies. Specifically, the lack of data on prior SARS-CoV-2 infection and the challenge in interpretation of VE by time since vaccination. The emergence of new variants may potentially have resulted in confounding by calendar time. VE estimates may also have been impacted by differences (e.g. risk factors, behaviours) between vaccinated and unvaccinated populations.

The average VE point estimates used in this study sourced from published studies included in the WHO live systematic review may not be an accurate estimation of VE within the population in Ireland due to differences in study populations, vaccination schedules, infection rates and different indirect impacts of vaccination within study populations compared with Ireland. There is therefore uncertainty attached to the estimation of VE in this study. A sensitivity analysis varying the VE by +/− 10% was conducted to address this uncertainty and to present a range around all reported estimates of vaccine impact. This follows approaches taken in other similar vaccine impact studies [19,20]. A further sensitivity analysis using average 95% CIs around each average VE point estimate was also undertaken. Additionally, there were limited VE estimates available against study outcomes for later vaccination doses; specifically, the third booster. VE estimates specific to Ireland were not calculated for this study due to small sample size available within the Sentinel GP surveillance network and the Severe Acute Respiratory Infection (SARI) surveillance system. Both networks are undergoing expansion currently to increase representativeness. These limitations in relation to VE data emphasise the importance of having national VE estimates, where possible, to use for future vaccine impact studies. Additionally, this study did not consider waning of VE over time and population vaccine coverage data by time since vaccination was not available. To further take uncertainty about VE into consideration, the latest reported VE estimates by time since vaccination for primary course and booster 1 were extracted from the literature. However, due to these uncertainties, VE may have been over or underestimated in this study.

Vaccine coverage data and VE estimates were not available by sex and therefore differences in vaccine impact by sex were not investigated in this study.

## Conclusion

During a period of Omicron variant dominance, the COVID-19 vaccination programme in Ireland averted symptomatic and severe cases of COVID-19 including deaths due to COVID-19. Vaccination therefore reduced morbidity and mortality in the population aged ≥ 50 years and protected population health and the healthcare system. In line with other international vaccine impact studies, the findings of this study emphasise the importance of high population vaccination coverage and the availability of effective vaccines and are relevant for informing ongoing COVID-19 booster vaccination programmes, planning future (autumn/winter) COVID-19 vaccination programmes, pandemic preparedness and communicating the rationale for and importance of vaccination in Ireland and internationally.

## Supplementary Data

469000 Supplementary Material

## References

[1] Department of Health. Strategic Approach for the Management of COVID-19 Preparedness for Autumn/Winter 2022/2023. Ireland: Department of Health; 2022.

[2] World Health Organization (WHO). Strategy considerations for severe acute respiratory syndrome coronavirus 2 (SARS-CoV-2) and other respiratory viruses in the WHO European Region during autumn and winter 2022/23. Geneva: WHO; 2022.

[3] Health Protection Surveillance Centre (HPSC). Summary of COVID-19 virus variants in Ireland, July 2023. Ireland: HPSC; 2023.

[4] Nyberg T Ferguson NM Nash SG Webster HH Flaxman S Andrews N COVID-19 Genomics UK (COG-UK) consortium . Comparative analysis of the risks of hospitalisation and death associated with SARS-CoV-2 omicron (B.1.1.529) and delta (B.1.617.2) variants in England: a cohort study. Lancet. 2022;399(10332):1303-12. 10.1016/S0140-6736(22)00462-7 35305296 PMC8926413

[5] Menni C May A Polidori L Louca P Wolf J Capdevila J COVID-19 vaccine waning and effectiveness and side-effects of boosters: a prospective community study from the ZOE COVID Study. Lancet Infect Dis. 2022;22(7):1002-10. 10.1016/S1473-3099(22)00146-3 35405090 PMC8993156

[6] National Immunisation Advisory Committee (NIAC). NIAC Immunisation Guidelines: Chapter 5a COVID-19. Ireland: Royal College of Physicians of Ireland; 2023.

[7] Government of Ireland. National COVID-19 Vaccination Programme: Strategy. Ireland: Government of Ireland; 2020.

[8] European Centre for Disease Prevention and Control (ECDC). Overview of the implementation of COVID-19 vaccination strategies and deployment plans in the EU/EEA. Stockholm: ECDC; 2023.

[9] Health Protection Surveillance Centre (HPSC). COVID-19 Vaccination Uptake in Ireland Weekly Report: Week 2 2022. Ireland: HPSC; 2022.

[10] National Immunisation Advisory Committee. Additional COVID-19 booster vaccination. Ireland: Royal College of Physicians of Ireland; 2022.

[11] European Centre for Disease Prevention and Control (ECDC). Preliminary public health considerations for COVID-19 vaccination strategies in the second half of 2022. Stockholm: ECDC; 2022.

[12] European Centre for Disease Prevention and Control (ECDC). Interim public health considerations for COVID-19 vaccination roll-out during 2023. Stockholm: ECDC; 2023.

[13] Lin DY Xu Y Gu Y Zeng D Wheeler B Young H Effectiveness of Bivalent Boosters against Severe Omicron Infection. N Engl J Med. 2023;388(8):764-6. 10.1056/NEJMc2215471 36734847 PMC9933929

[14] Arbel R Peretz A Sergienko R Friger M Beckenstein T Duskin-Bitan H Effectiveness of a bivalent mRNA vaccine booster dose to prevent severe COVID-19 outcomes: a retrospective cohort study. Lancet Infect Dis. 2023;23(8):914-21. 10.1016/S1473-3099(23)00122-6 37062302 PMC10156150

[15] Health Service Executive (HSE) National Immunisation Office. Table of recommended groups for COVID-19 autumn booster and flu vaccine. Ireland: HSE; 2023.

[16] Health Protection Surveillance Centre (HPSC). COVID-19 Vaccination Uptake in Ireland Weekly Report: Week 24 2023. Ireland: HPSC; 2023.

[17] Health Protection Surveillance Centre (HPSC). COVID-19 Vaccination Uptake in Ireland Weekly Report: Week 16 2023. Ireland: HPSC; 2023.

[18] Machado A Kislaya I Larrauri A Matias Dias C Nunes B . Impact of national influenza vaccination strategy in severe influenza outcomes among the high-risk Portuguese population. BMC Public Health. 2019;19(1):1690. 10.1186/s12889-019-7958-8 31842831 PMC6916191

[19] Sacco C Mateo-Urdiales A Petrone D Spuri M Fabiani M Vescio MF Italian Integrated Surveillance of COVID-19 study group . Estimating averted COVID-19 cases, hospitalisations, intensive care unit admissions and deaths by COVID-19 vaccination, Italy, January-September 2021. Euro Surveill. 2021;26(47):2101001. 10.2807/1560-7917.ES.2021.26.47.2101001 34823637 PMC8619872

[20] Meslé MM Brown J Mook P Hagan J Pastore R Bundle N Estimated number of deaths directly averted in people 60 years and older as a result of COVID-19 vaccination in the WHO European Region, December 2020 to November 2021. Euro Surveill. 2021;26(47):2101021. 10.2807/1560-7917.ES.2021.26.47.2101021 34823641 PMC8619871

[21] Health Service Executive (HSE). Winter Plan October 2022-March 2023. Ireland: HSE; 2022.

[22] International Vaccine Access Centre Johns Hopkins Bloomberg School of Public Health and World Health Organisation (WHO) and Coalition for Epidemic Preparedness Innovations. Results of COVID-19 Vaccine Effectiveness & Impact Studies: An Ongoing Systematic Review: Methods. Geneva: WHO; 2023.

[23] VIEW-Hub . Available from: https://view-hub.org/resources

[24] Solante R Alvarez-Moreno C Burhan E Chariyalertsak S Chiu NC Chuenkitmongkol S Expert review of global real-world data on COVID-19 vaccine booster effectiveness and safety during the omicron-dominant phase of the pandemic. Expert Rev Vaccines. 2023;22(1):1-16. 10.1080/14760584.2023.2143347 36330971

[25] Central Statistics Office (CSO). Preliminary Census 2022 results. Dublin: CSO; 2022.

[26] R Core Team. (2013). A language and environment for statistical computing. Vienna, Austria: R Foundation for Statistical Computing; 2013.

[27] Tamandjou Tchuem CR Auvigne V Vaux S Montagnat C Paireau J Monnier Besnard S Vaccine effectiveness and duration of protection of COVID-19 mRNA vaccines against Delta and Omicron BA.1 symptomatic and severe COVID-19 outcomes in adults aged 50 years and over in France. Vaccine. 2023;41(13):2280-8. 10.1016/j.vaccine.2023.02.062 36870880 PMC9968619

[28] Kirsebom FCM Andrews N Sachdeva R Stowe J Ramsay M Lopez Bernal J . Effectiveness of ChAdOx1-S COVID-19 booster vaccination against the Omicron and Delta variants in England. Nat Commun. 2022;13(1):7688. 10.1038/s41467-022-35168-7 36509743 PMC9744366

[29] Link-Gelles R Ciesla AA Fleming-Dutra KE Smith ZR Britton A Wiegand RE Effectiveness of Bivalent mRNA Vaccines in Preventing Symptomatic SARS-CoV-2 Infection - Increasing Community Access to Testing Program, United States, September-November 2022. MMWR Morb Mortal Wkly Rep. 2022;71(48):1526-30. 10.15585/mmwr.mm7148e1 36454688 PMC9721148

[30] Thompson MG Natarajan K Irving SA Rowley EA Griggs EP Gaglani M Effectiveness of a Third Dose of mRNA Vaccines Against COVID-19-Associated Emergency Department and Urgent Care Encounters and Hospitalizations Among Adults During Periods of Delta and Omicron Variant Predominance - VISION Network, 10 States, August 2021-January 2022. MMWR Morb Mortal Wkly Rep. 2022;71(4):139-45. 10.15585/mmwr.mm7104e3 35085224 PMC9351525

[31] Ferdinands JM Rao S Dixon BE Mitchell PK DeSilva MB Irving SA Waning 2-Dose and 3-Dose Effectiveness of mRNA Vaccines Against COVID-19-Associated Emergency Department and Urgent Care Encounters and Hospitalizations Among Adults During Periods of Delta and Omicron Variant Predominance - VISION Network, 10 States, August 2021-January 2022. MMWR Morb Mortal Wkly Rep. 2022;71(7):255-63. 10.15585/mmwr.mm7107e2 35176007 PMC8853475

[32] Natarajan K Prasad N Dascomb K Irving SA Yang DH Gaglani M Effectiveness of Homologous and Heterologous COVID-19 Booster Doses Following 1 Ad.26.COV2.S (Janssen [Johnson & Johnson]) Vaccine Dose Against COVID-19-Associated Emergency Department and Urgent Care Encounters and Hospitalizations Among Adults - VISION Network, 10 States, December 2021-March 2022. MMWR Morb Mortal Wkly Rep. 2022;71(13):495-502. 10.15585/mmwr.mm7113e2 35358170 PMC8979598

[33] Tartof SY Slezak JM Puzniak L Hong V Frankland TB Xie F Effectiveness and durability of BNT162b2 vaccine against hospital and emergency department admissions due to SARS-CoV-2 omicron sub-lineages BA.1 and BA.2 in a large health system in the USA: a test-negative, case-control study. Lancet Respir Med. 2023;11(2):176-87. 10.1016/S2213-2600(22)00354-X 36216013 PMC9765328

[34] Link-Gelles R Levy ME Gaglani M Irving SA Stockwell M Dascomb K Effectiveness of 2, 3, and 4 COVID-19 mRNA Vaccine Doses Among Immunocompetent Adults During Periods when SARS-CoV-2 Omicron BA.1 and BA.2/BA.2.12.1 Sublineages Predominated - VISION Network, 10 States, December 2021-June 2022. MMWR Morb Mortal Wkly Rep. 2022;71(29):931-9. 10.15585/mmwr.mm7129e1 35862287 PMC9310634

[35] Ferdinands JM Rao S Dixon BE Mitchell PK DeSilva MB Irving SA Waning of vaccine effectiveness against moderate and severe covid-19 among adults in the US from the VISION network: test negative, case-control study. BMJ. 2022;379:e072141. 10.1136/bmj-2022-072141 36191948 PMC9527398

[36] Link-Gelles R Levy ME Natarajan K Reese SE Naleway AL Grannis SJ Estimation of COVID-19 mRNA Vaccine Effectiveness and COVID-19 Illness and Severity by Vaccination Status During Omicron BA.4 and BA.5 Sublineage Periods. JAMA Netw Open. 2023;6(3):e232598. 10.1001/jamanetworkopen.2023.2598 36920396 PMC10018321

[37] Tartof SY Slezak JM Puzniak L Hong V Frankland TB Ackerson BK BNT162b2 vaccine effectiveness against SARS-CoV-2 omicron BA.4 and BA.5. Lancet Infect Dis. 2022;22(12):1663-5. 10.1016/S1473-3099(22)00692-2 36306800 PMC9597567

[38] Bozio CH Butterfield KA Briggs Hagen M Grannis S Drawz P Hartmann E Protection From COVID-19 mRNA Vaccination and Prior SARS-CoV-2 Infection Against COVID-19-Associated Encounters in Adults During Delta and Omicron Predominance. J Infect Dis. 2023;227(12):1348-63. 10.1093/infdis/jiad040 36806690 PMC13259584

[39] Baum U Poukka E Leino T Kilpi T Nohynek H Palmu AA . High vaccine effectiveness against severe COVID-19 in the elderly in Finland before and after the emergence of Omicron. BMC Infect Dis. 2022;22(1):816. 10.1186/s12879-022-07814-4 36335289 PMC9636823

[40] Carazo S, Skowronski DM, Brisson M, Sauvageau C, Brousseau N, Fafard J, et al. Prior infection- and/or vaccine-induced protection against Omicron BA.1, BA.2 and BA.4/BA.5-related hospitalisations in older adults: a test-negative case-control study in Quebec, Canada. medRxiv: the preprint server for health sciences. 2022:2022.12.21.22283740. 10.1101/2022.12.21.22283740 10.1101/2022.12.21.22283740

[41] Stowe J Andrews N Kirsebom F Ramsay M Bernal JL . Effectiveness of COVID-19 vaccines against Omicron and Delta hospitalisation, a test negative case-control study. Nat Commun. 2022;13(1):5736. 10.1038/s41467-022-33378-7 36180428 PMC9523190

[42] Sharma A Oda G Holodniy M . Effectiveness of Messenger RNA-based Vaccines During the Emergence of the Severe Acute Respiratory Syndrome Coronavirus 2 Omicron Variant. Clin Infect Dis. 2022;75(12):2186-92. 10.1093/cid/ciac325 35475889 PMC9129111

[43] Wan EYF Mok AHY Yan VKC Chan CIY Wang B Lai FTT Effectiveness of BNT162b2 and CoronaVac vaccinations against SARS-CoV-2 omicron infection in people aged 60 years or above: a case-control study. J Travel Med. 2022;29(8):taac119. 10.1093/jtm/taac119 36250571 PMC9619717

[44] Young-Xu Y Zwain GM Izurieta HS Korves C Powell EI Smith J Effectiveness of mRNA COVID-19 vaccines against Omicron and Delta variants in a matched test-negative case-control study among US veterans. BMJ Open. 2022;12(8):e063935. 10.1136/bmjopen-2022-063935 35922100 PMC9352567

[45] Gram MA Emborg HD Schelde AB Friis NU Nielsen KF Moustsen-Helms IR Vaccine effectiveness against SARS-CoV-2 infection or COVID-19 hospitalization with the Alpha, Delta, or Omicron SARS-CoV-2 variant: A nationwide Danish cohort study. PLoS Med. 2022;19(9):e1003992. 10.1371/journal.pmed.1003992 36048766 PMC9436060

[46] Kislaya I Machado A Magalhães S Rodrigues AP Franco R Leite PP COVID-19 mRNA vaccine effectiveness (second and first booster dose) against hospitalisation and death during Omicron BA.5 circulation: cohort study based on electronic health records, Portugal, May to July 2022. Euro Surveill. 2022;27(37):2200697. 10.2807/1560-7917.ES.2022.27.37.2200697 36111555 PMC9479470

[47] Yan VKC Wan EYF Ye X Mok AHY Lai FTT Chui CSL Effectiveness of BNT162b2 and CoronaVac vaccinations against mortality and severe complications after SARS-CoV-2 Omicron BA.2 infection: a case-control study. Emerg Microbes Infect. 2022;11(1):2304-14. 10.1080/22221751.2022.2114854 35980089 PMC9553171

[48] Park SK Choe YJ Jang EJ Kim RK Lee SW Kwon GY Effectiveness of Heterologous COVID-19 Vaccine Booster in Korean Elderly Population, 2022. J Korean Med Sci. 2023;38(19):e143. 10.3346/jkms.2023.38.e143 37191847 PMC10186072

[49] Shrotri M Krutikov M Palmer T Giddings R Azmi B Subbarao S Vaccine effectiveness of the first dose of ChAdOx1 nCoV-19 and BNT162b2 against SARS-CoV-2 infection in residents of long-term care facilities in England (VIVALDI): a prospective cohort study. Lancet Infect Dis. 2021;21(11):1529-38. 10.1016/S1473-3099(21)00289-9 34174193 PMC8221738

[50] Liu B Stepien S Dobbins T Gidding H Henry D Korda R Effectiveness of COVID-19 vaccination against COVID-19 specific and all-cause mortality in older Australians: a population based study. Lancet Reg Health West Pac. 2023;40:100928. 10.1016/j.lanwpc.2023.100928 37854458 PMC10579525

[51] Stamm TA Partheymüller J Mosor E Ritschl V Kritzinger S Alunno A Determinants of COVID-19 vaccine fatigue. Nat Med. 2023;29(5):1164-71. 10.1038/s41591-023-02282-y 36973410 PMC10202806

[52] Menegale F Manica M Zardini A Guzzetta G Marziano V d’Andrea V Evaluation of Waning of SARS-CoV-2 Vaccine-Induced Immunity: A Systematic Review and Meta-analysis. JAMA Netw Open. 2023;6(5):e2310650. 10.1001/jamanetworkopen.2023.10650 37133863 PMC10157431

[53] European Centre for Disease Prevention and Control (ECDC). Country overview report: week 31 2023. Stockholm: ECDC; 2023.

[54] McKeown D McCourt A Hendrick L O’Farrell A Donohue F Grabowsky L COVID-19 incidence and outcome by affluence/deprivation across three pandemic waves in Ireland: A retrospective cohort study using routinely collected data. PLoS One. 2023;18(7):e0287636. 10.1371/journal.pone.0287636 37478117 PMC10361474

[55] Department of Health and Health Service Executive. Impact of COVID on the health service. Ireland: Department of Health; 2021.

[56] Health Service Executive (HSE). National Adult Critical Care Capacity - Census 2021 Report. Ireland: HSE; 2021.

[57] Watson OJ Barnsley G Toor J Hogan AB Winskill P Ghani AC . Global impact of the first year of COVID-19 vaccination: a mathematical modelling study. Lancet Infect Dis. 2022;22(9):1293-302. 10.1016/S1473-3099(22)00320-6 35753318 PMC9225255

