# Supplementary material for: The impact of the COVID-19 vaccination programme on symptomatic and severe SARS-CoV-2 infection during a period of Omicron variant dominance in Ireland, December 2021 to March 2023
Source: Euro Surveill. 2024 Jul 11;29(28):2300697. doi: 10.2807/1560-7917.ES.2024.29.28.2300697 (PMC11241852; doi:10.2807/1560-7917.ES.2024.29.28.2300697)
Supplement: Supplementary Material [file 2300697_SupplementaryMaterial.pdf]

## Supplementary materials

This supplementary material is hosted by Eurosurveillance as supporting information alongside the article **The impact of the COVID-19 vaccination programme on symptomatic and severe SARS-CoV-2 infection during a period of Omicron variant dominance in Ireland, December 2021 to March 2023** on behalf of the authors, who remain responsible for the accuracy and appropriateness of the content. The same standards for ethics, copyright, attributions and permissions as for the article apply. Supplements are not edited by Eurosurveillance and the journal is not responsible for the maintenance of any links or email addresses provided therein.

### The rollout of the COVID-19 vaccination programme in Ireland

The COVID-19 vaccination programme commenced in Ireland in December 2020. Vaccines were administered within the public healthcare system and were free of charge. Vaccines have been administered over time according to the National Immunisation Advisory Committee (NIAC) guidelines on prioritisation for groups at highest risk of complications from SARS-CoV-2 infection, including those in older age groups and those at high risk of complications of infection due to underlying medical conditions or immunocompromise[1].

#### *COVID-19 vaccination coverage in Ireland*

High COVID-19 vaccination coverage was achieved in Ireland for the primary vaccination series and first booster dose with over 97% uptake for the primary vaccination course and over 90% uptake for first booster dose among those aged 50  years and older by week 24 2023 (Table S1)[2]. The weekly cumulative vaccination uptake in those aged 50 years and older during this study period (Week 53 2020 to week 12 2023) in Ireland is shown in Figure S1.

**Table S1 COVID-19 vaccination uptake of eligible population* by age group and vaccination status, Ireland: Week 24 2023, Ireland**

| Age group | Primary course | Booster 1 | Booster 2 | Booster 3 | Booster 4 |
|---|---|---|---|---|---|
| 70+ years | 99.9 | 98.2 | 84.1 | 61.7 | 29.2 |
| 65 + years | 99.6 | 97.5 | 80.9 | 55.5 | 21.7 |
| 60 + years | 98.6 | 96.5 | 74.4 | - | - |
| 50 + years | 97.3 | 93.5 | 61.5 | - | - |
| 18 + years | 91.9 | 81.1 | 34.5 | - | - |
| 12-17 years | 65.9 | 33.0 | 0.7 | - | - |
| 12 + years | 89.3 | 77.5 | 31.1 | - | - |
| 5+ years | 82.3 | 75.6 | - | - | - |
| 6 months + | 78.1 | - | - | - | - |

**Percentage booster uptake is based on the proportion of those who have completed their primary course treatment*

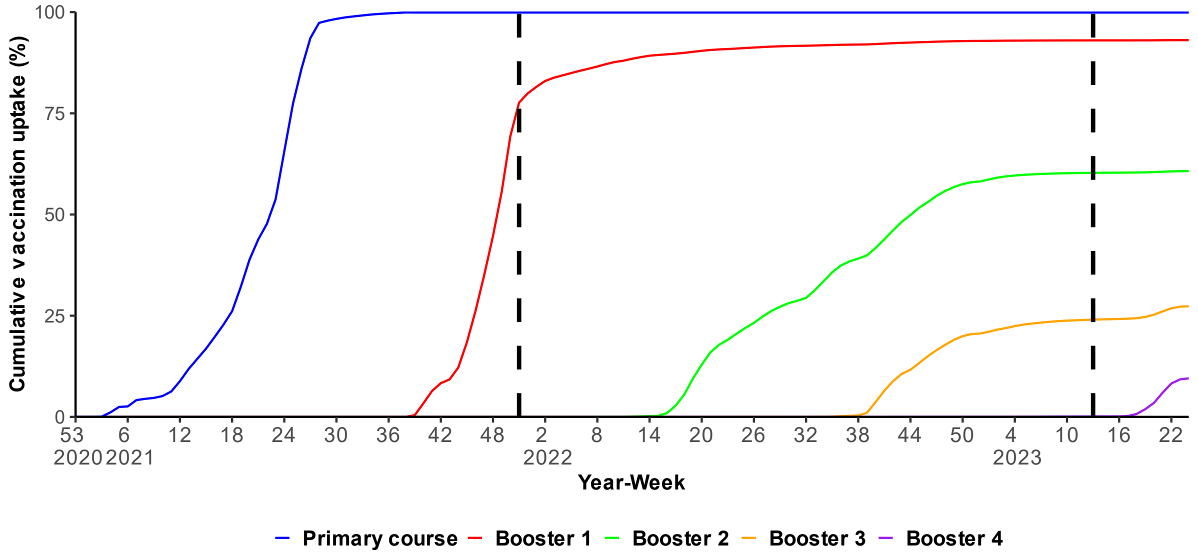


*Dashed lines indicate the beginning and end of the study period*

**Figure S1 Weekly cumulative vaccination uptake in those aged 50 years and older: Week 53 2020 to week 24 2023, Ireland**

***COVID-19 vaccines used in Ireland***

Three different categories of European Medicines Agency (EMA) authorised COVID-19 vaccines that have been used in Ireland are[1]:

- mRNA: Pfizer/BioNTech (Comirnaty) and Moderna (Spikevax)
  - Bivalent mRNA vaccines (Spikevax bivalent Original/Omicron BA.1, Comirnaty Original/Omicron BA4-5) have been available in Ireland since September 2022
- Viral vector: AstraZeneca (Vaxevria) and COVID-19 vaccine Janssen (JCOVDEN)
- Protein subunit: Novavax (Nuvaxovid)

From December 2020 for the rollout of the primary immunisation course in Ireland, both mRNA and viral vector vaccines were used in Ireland. As the rollout progressed in 2021, mRNA vaccines were preferentially used in those aged under 50 years for the primary immunisation course, due to risk of Vaccine-Associated Thrombosis with Thrombocytopenia Syndrome (TTS)[3]. The rollout of the first COVID-19 booster began in Ireland in September 2021 and for this and for subsequent booster vaccination campaigns in Ireland, mRNA vaccines have been preferentially recommended[1].

For the 2023/2024 autumn/winter COVID-19 vaccination programme, the vaccines available in Ireland are:

- Pfizer/BioNTech (Comirnaty) and
- Nuvaxovid

The Pfizer/BioNTech (Comirnaty) is the recommended vaccine for the majority of the population.

***Population groups recommended to receive COVID-19 vaccination in Ireland***

Vaccination allocation sequencing in 2020 at the commencement of the COVID-19 vaccination programme in Ireland was as follows[4].

**Table S2 Prioritisation groups for COVID-19 vaccination, December 2020, Ireland[4]**

| **Group** |
|---|
| Adults aged ≥65 years who are residents of long-term care facilities. |
| Frontline healthcare workers (HCWs) in direct patient contact roles (including vaccinators) or who risk exposure to bodily fluids or aerosols. |
| Aged 70 and older in the following order: 85 and older 80-84 75-79 70-74. |
| Other HCWs not in direct patient contact. |
| Aged 65-69. Prioritise those with medical conditions which put them at high risk of severe disease. |
| Key workers (to be further refined) including those providing services essential to the vaccination programme e.g. logistical support. |
| Aged 18-64 years with medical conditions which put them at high risk of severe disease. |
| Residents of long-term care facilities aged 18-64. |
| Aged 18-64 years living working in crowded accommodation where self-isolation and social distancing is difficult to maintain. |
| Key workers in essential jobs who cannot avoid a high risk of exposure to COVID-19. They include workers in the food supply system, public and commercial transport and other vital services. |
| Those who are essential to education and who face disease exposure - primary and second level school staff, special needs assistants, childcare workers, maintenance workers, school bus drivers etc. |
| Aged 55-64 years. |
| Those in occupations important to the functioning of society, e.g., third level institutions, entertainment and goods-producing industries who work in settings where protective measures can be followed without much difficulty. |
| Aged 18-54 years who did not have access to the vaccine in prior phases |
| Children, adolescents up to 18 years and pregnant women |

The allocation groups were reviewed and refined by the NIAC in Ireland as the vaccination programme rollout progressed in Ireland, informed by emerging evidence including vaccine effectiveness, the factors associated with severe infection and the evolving epidemiological situation and emerging COVID-19 variants in Ireland and internationally. The vaccination priority groups for COVID-19 vaccination in the for the COVID-19 autumn and winter vaccination programme in 2023/2024 is outlined in Table S3. Adapted from the Health Service Executive (HSE) National Immunisation Office table of recommendations in November 2023[5]. Available at:

https://www.hse.ie/eng/health/immunisation/hcpinfo/covid19vaccineinfo4hps/table-of-recommended-groups-for-covid19-autumn-booster-and-flu-vaccine.pdf

**Table S3: Recommended groups for COVID-19 autumn booster, Ireland, 2023**

| Age and cohort | COVID-19 autumn booster |
|---|---|
| 65 and older | Recommended for all (at least 6 months since last booster dose of SARS-CoV-2 infection) |
| 50 to 64 years | Recommended for all (at least 6 months since last booster dose of SARS-CoV-2 infection) |
| 18 to 49 years | Recommended for:<br>Those with immunocompromise associated with a suboptimal response to vaccination (at least 6 months since last booster dose of SARS-CoV-2 infection)<br><br>Those with medical conditions associated with a higher risk of COVID-19 hospitalisation, severe disease or death (at least 9 months since last booster dose of SARS-CoV-2 infection) |
| 5 to 17 years | Recommended for:<br>Those with immunocompromise associated with a suboptimal response to vaccination (at least 6 months since last booster dose of SARS-CoV-2 infection)<br><br>Those with medical conditions associated with a higher risk of COVID-19 hospitalisation, severe disease or death (at least 9 months since last booster dose of SARS-CoV-2 infection) |
| Healthcare workers | Recommended for all |
| Pregnancy | A COVID-19 booster dose is recommended at least once in pregnancy.<br><br>If no previous booster: Booster recommended at least 4 months after last COVID-19 vaccine does or SARS-CoV-2 infection<br>If received a booster prior to pregnancy booster recommended at least 6 months after last COVID-19 vaccine dose or SARS-CoV-2 infection |

Additional information on recommended groups for the COVID-19 vaccination programme in Ireland during the 2023/2024 season is available in the NIAC guidelines for Ireland[1]. Available at

https://www.rcpi.ie/Healthcare-Leadership/NIAC/Immunisation-Guidelines-for-Ireland

***Definition of vaccination status used for COVID-19 vaccination coverage calculations[2]***

*Primary series course completed*

This is two doses of a two-dose vaccine course or one dose of a single Janssen (JCOVDEN) vaccine, including those who are immunocompromised (as per the NIAC guidelines) and have received an extra, third (primary) dose as an extended primary course. Those with one dose schedules are considered fully vaccinated 14 days or more after receipt of the second dose. Those with two dose schedules are considered fully vaccinated 14 days or more after receipt of the dose. Those with a two-dose schedule are considered fully vaccinated 7 days of more after receipt of the third dose.

*First booster dose*

Completed a primary series course and have received a first booster dose. Considered to be vaccinated with a first booster 7 days or more after receipt of the first booster dose.

*Second booster dose*

Completed a primary series course and have received a second booster dose. Considered to be vaccinated with a second booster 7 days or more after receipt of the second booster dose.

*Third booster dose*

Completed a primary series course and have received a third booster dose. Considered to be vaccinated with a third booster 7 days or more after receipt of the third booster dose.

*Fourth booster dose*

Completed a primary series course and have received a fourth booster dose. Considered to be vaccinated with a third booster 7 days or more after receipt of the fourth booster dose.

*Not vaccinated*

No record of COVID-19 vaccination.

**Overview of COVID-19 testing strategies during Omicron dominance in Ireland: December 2021 to 1st April 2023**

Testing policies can affect the epidemiology of notified cases In Ireland, in winter 2021/2022, there was ongoing polymerase chain reaction (PCR) testing for COVID-19 within the population both clinically directed and walk-in testing for symptomatic individuals. Testing was recommended for vaccinated individuals who were symptomatic only and there was a serial testing programme for vulnerable groups e.g. nursing home residents. In early 2022, Ireland underwent a transition from the containment phase of the pandemic to a mitigation phase[6]. From February 2022 (epidemiological week 9), testing was not recommended routinely for all who were symptomatic however and was restricted to those in specific risk groups e.g. those aged 55 years and older who had not had a COVID-19 booster vaccine dose, healthcare workers and those with a high-risk medical condition or immunocompromise (defined in NIAC guidelines)[1]. Guidelines for testing strategies were stable over the winter period in 2022/2023[6].

National COVID-19 testing policies changed to a clinically driven model in April 2023. Currently, COVID-19 testing is available based on clinical assessment by GPs to inform the diagnosis and management of individual patients and additionally, testing is arranged by Public Health when indicated, for example, in the context of outbreaks[7]. This change was undertaken in conjunction with a strengthening of respiratory virus surveillance programmes. This strengthened surveillance includes GP sentinel surveillance, serosurveillance, excess mortality surveillance, Severe Acute Respiratory Infection (SARI) surveillance and a national whole genome sequencing programme.

**Overview of non-pharmaceutical interventions during Omicron dominance in Ireland: December 2021 to 1st April 2023**

In Ireland in winter 2021/2022, extensive non-pharmaceutical interventions (NPIs) were in place including a recommendation for face masks/coverings in all indoor settings, social distancing and reduced opening hours and capacity for indoor events and restricted movement advice for all close contacts regardless of vaccination status. Guidance changed over the winter period with greater restrictions recommended over the Christmas period which were subsequently relaxed and the requirement to implement public health restrictive measures e.g. early closing time for businesses and capacity restrictions such were removed from 1st April 2022[6]. However, specific NPIs continued to be recommended and during winter 2022/2023 there were a number of continued NPIs recommended, including[6]:

- Face masks/coverings were recommended on public transport and in healthcare settings.

- Personal protective measures were recommended e.g., staying at home when symptomatic and hand and respiratory hygiene.
- Mask wearing was also advised based on individual risk assessment.
- Sectors were advised to continue to implement NPIs as appropriate to each sector.
- Significant enhancement of communication on ventilation and air filtration, including necessary guidance and supports was recommended.

**Study specific inclusion criteria**

The literature search strategy for the WHO's live ongoing systematic review and the inclusion criteria are publicly available at https://view-hub.org/resources.

The study specific inclusion criteria for this national vaccine impact study were:

- VE must have been reported against the specific study outcomes
- Studies should have reported VE data on adults aged 50 years and older
- VE estimates must be reported for the Omicron variant specifically; estimates for the Omicron variant and its sublineages were included
- The study must have been conducted in adult populations in the general population against unvaccinated controls and report absolute VE
- The study did not necessarily consider underlying medical conditions or have taken place in specific subgroups e.g., those in long term care facilities only
- The study must have reported VE for European Medicines Agency (EMA) approved vaccines that have been administered as part of the COVID-19 vaccination programme in Ireland (supplementary materials)
- The study should preferentially have been conducted in WHO Europe countries, USA or Canada however if VE estimates were limited or not available against a specific outcome (e.g., ICU admission), data from studies conducted in Australia, New Zealand South Korea and Hong Kong were included, if all other inclusion criteria were met
- Definitions of fully vaccinated and booster doses must align with national guidelines in Ireland (supplementary materials)

When extracting VE estimates from studies that meet the inclusion criteria, the following methodology were used. This methodology is informed by the WHO methodology for extracting VE estimates[8], by other studies that have used IVAC data[9], and by consultation with colleagues working on vaccine impact studies in WHO. These criteria were:

- When VE estimates were available for subgroups e.g., those who received a BNT162b2 (Pfizer) or mRNA-1273 (Moderna) vaccine or a heterologous course for primary course or booster vaccination, subgroups were treated as separate samples.
- When multiple follow up VE estimates were reported at time points since vaccination in the same study, for the primary course and first booster vaccination, the latest reported VE estimates were used. This was because the coverage of the primary course at the beginning of the study period was 99.9% and the coverage of booster 1 vaccinations was 78% (Figure 1).

Therefore, those with these vaccination statuses would likely have had a considerable time interval from last vaccination.

- The rollout of second and third booster doses began during the study period (Figure 2). Therefore, those with this vaccination status were vaccinated during the study period and would likely have had a shorter interval from last vaccination. Therefore, for these vaccination statuses, the earliest reported VE estimates (after 14 days post-vaccination) were extracted.
- When VE estimates for the age group of interest (50 years and older) were not reported for a specific outcome, estimates from the closest age group were extracted.
- If no data were available on VE of third booster doses against a specific outcome e.g., ICU admission, the VE of the second booster were used as a proxy estimate.

## Sensitivity analyses

**Table S4 Observed, averted and expected outcomes and prevented fraction (PF) in those aged 50 years and older: Week 51 2021 to week 12 2023, Ireland: Sensitivity analysis varying the definition of a COVID-19 death**

| Outcome[1,2] | Observed | Averted | Averted range[3] | Expected | Expected range[3] | Observed rate[4] | Expected rate[4] | Expected rate range[34] | PF[5] | PF range[3,5] |
|---|---|---|---|---|---|---|---|---|---|---|
| Death (Primary analysis) | 2,429 | 15,985 | 8,031 – 61,814 | 18,414 | 10,460 – 64,243 | 143.2 | 1,085.6 | 616.7 – 3,787.6 | 0.87 | 0.77 - 0.96 |
| Death (Sensitivity analysis) | 2,397 | 15,785 | 7,928 – 61,106 | 18,182 | 10,325 – 63,503 | 141.3 | 1,072.0 | 608.8 – 3,744 | 0.87 | 0.77 - 0.96 |

[1] 3 week rolling average

[2] estimates based varying definition of a COVID-19 death (described in manuscript)

[3] Range calculated by varying average VE point estimates by +/- 10%

[4] per 100,000 population

[5] PF = Prevented fraction

**Table S5 Observed, averted and expected outcomes and prevented fraction (PF) in those aged 50 years and older: Week 51 2021 to week 12 2023, Ireland: Sensitivity analysis using counts of outcomes rather than 3 week rolling averages**

| Outcome | Observed | Averted | Averted range[1] | Expected | Expected range[1] | Observed rate[2] | Expected rate[2] | Expected rate range[1,2] | PF[3] | PF range[1,3] |
|---|---|---|---|---|---|---|---|---|---|---|
| Symptomatic case[4] | 86,098 | 48,289 | 30,132 – 73,176 | 134,387 | 116,230 – 159,274 | 5,076.1 | 7,923.0 | 6,852.6 – 9,390.3 | 0.36 | 0.26 - 0.46 |
| Emergency Department presentation[5] | 8,342 | 9,439 | 6,313 – 14,265 | 17,781 | 14,655 – 22,607 | 491.8 | 1,048.3 | 864 – 1,332.9 | 0.53 | 0.43 - 0.63 |
| Hospitalisation | 24,548 | 102,043 | 58,683 – 245,785 | 126,591 | 83,231 – 270,333 | 1,447.3 | 7,463.4 | 4,907.1 – 15,938.0 | 0.81 | 0.71 - 0.91 |
| Intensive Care Unit admission | 389 | 3,277 | 1,488 – 15,675 | 3,666 | 1,877 – 16,064 | 22.9 | 216.1 | 110.7 - 947.1 | 0.89 | 0.79 - 0.98 |
| Death | 2,422 | 15,947 | 8,010 – 61,722 | 18,369 | 10,432 – 64,144 | 142.8 | 1,083.0 | 615.1 – 3,781.8 | 0.87 | 0.77 - 0.96 |

[1] Range calculated by varying average VE point estimates by +/- 10%

[2] Per 100,000 population

[3] Prevented fraction (with range)

[4] Symptomatic COVID-19 case presenting to primary care or community testing centre

[5] Emergency Department presentation excluded cases subsequently hospitalised or who were admitted to an intensive care unit or who died

**Table S6 Averted outcomes in those aged 50 years and older by vaccination status: Week 51 2021 to week 12 2023, Ireland: Sensitivity analysis using counts of outcomes rather than 3 week rolling averages**

| Outcome | Observed | Averted | Averted range[1] | PC[2] | PC range[1,2] | B1[3] | B1 range[1,3] | B2[4] | B2 range[1,4] | B3[5] | B3 range[1,5] |
|---|---|---|---|---|---|---|---|---|---|---|---|
| Symptomatic case[6] | 86,098 | 48,289 | 30,132 – 73,176 | 3,619 | 1,564 – 6,438 | 42,324 | 140 - 420 | 2,086 | 990 – 3,565 | 260 | 140 - 420 |
| Emergency Department presentation[7] | 8,342 | 9,439 | 6,313 – 14,265 | 623 | 337 – 1,062 | 7,029 | 270 - 591 | 1,392 | 940 – 2,102 | 394 | 270 - 591 |
| Hospitalisation | 24,548 | 102,043 | 58,683 – 245,785 | 8,083 | 4,421 – 20,529 | 73,258 | 2,667 – 8,861 | 16,276 | 9,575 – 35,020 | 4,425 | 2,667 – 8,861 |
| Intensive Care Unit admission | 389 | 3,277 | 1,488 – 15,675 | 312 | 142 – 1,499 | 2,134 | 94 – 1,055 | 606 | 258 – 2,899 | 225 | 94 – 1,055 |
| Death | 2,422 | 15,947 | 8,010 – 61,722 | 1,474 | 718 – 5,913 | 11,459 | 360 – 2,223 | 2,328 | 1,199 – 8,133 | 686 | 360 – 2,223 |

[1] Range calculated by varying average VE point estimates by +/- 10%

[2] PC = Outcome averted among those who had primary vaccination course only with range

[3] B1 = Outcome averted among those who had primary vaccination course and booster 1 only with range

[4] B2 = Outcome averted among those who had primary vaccination course, booster 1 and booster 2 only with range

[5] B3 = Outcome averted among those who had primary vaccination course, booster 1, booster 2 and booster 3 only with range

[6] Symptomatic COVID-19 case presenting to primary care or community testing centre

[7] Emergency Department presentation excluded cases subsequently hospitalised or who were admitted to an intensive care unit or who died

### Sensitivity analysis varying the range around the average point VE estimates

Average VE point estimates for the Omicron variant calculated against symptomatic infection/cases[10-12], ED presentation[13-21], hospitalisation[11,19,22-29], ICU admission[24,26,30,31], and death[25-27,29-33] are shown in Table S7. Average 95% CIs around each estimate used for a sensitivity analysis is includes as supplementary materials.

**Table S7 Average COVID-19 vaccine effectiveness point estimates by outcome[34]**

*Source: https://view-hub.org/resources*

| Vaccine effectiveness (%) by outcome | Primary course | | | Booster 1 | | | Booster 2 | | | Booster 3 | | |
|---|---|---|---|---|---|---|---|---|---|---|---|---|
| | VE | LCI[a] | UCI[b] | VE | LCI[a] | UCI[b] | VE | LCI[a] | UCI[b] | VE | LCI[a] | UCI[b] |
| **Symptomatic infection/cases*** | 20 | 16 | 24 | 40 | 27 | 50 | 22 | 13 | 31 | 26 | 18 | 32 |
| **Emergency Department presentation** | 29 | 21 | 36 | 56 | 49 | 62 | 57 | 42 | 67 | 62 | 47 | 73 |
| **Hospitalisation** | 62 | 43 | 75 | 86 | 79 | 90 | 73 | 59 | 82 | 73 | 67 | 78 |
| **Intensive Care Unit admission** | 76 | 67 | 81 | 90 | 82 | 94 | 94 | 89 | 96 | 94 | 89 | 96 |
| **Death** | 72 | 68 | 80 | 90 | 91 | 96 | 84 | 69 | 90 | 84 | 69 | 90 |

*[a]Lower 95% confidence interval [b]Upper 95% confidence interval*

**Symptomatic infection/cases were not hospitalised and were not classified as having severe infection*

The vaccine impact measures calculated using the estimates in Table S7 to create ranges (using average 95% confidence intervals) around the measures of impact are shown in Table S8 and Table S9.

**Table S8 Observed, averted and expected outcomes and prevented fraction (PF) in those aged 50 years and older: Week 51 2021 to week 12 2023, Ireland: Sensitivity analysis**

| Outcome[1] | Observed | Averted | Averted range[2] | Expected | Expected range[2] | Observed rate[3] | Expected rate[3] | Expected rate range[2] | PF[4] | PF range[2,4] |
|---|---|---|---|---|---|---|---|---|---|---|
| Symptomatic case[5] | 86,570 | 48,551 | 27,901 – 70,935 | 135,121 | 114,471 – 157,505 | 5,103.9 | 7,966.3 | 6,748.9 – 9,286 | 0.36 | 0.24 - 0.45 |
| ED presentation[6] | 8,422 | 9,517 | 6,771 – 12,616 | 17,939 | 15,193 – 21,038 | 496.5 | 1,057.6 | 896- 1,240 | 0.53 | 0.45 - 0.60 |
| Hospitalisation | 24,578 | 102,160 | 60,594 – 157,142 | 126,738 | 85,172 – 181,720 | 1,449.0 | 7,472.1 | 5,022 – 10,714 | 0.81 | 0.71 - 0.86 |
| ICU admission[7] | 392 | 3,303 | 1,782 – 5,222 | 3,695 | 2,174 – 5,614 | 23.1 | 217.8 | 128 - 331 | 0.89 | 0.82 - 0.93 |
| Death | 2,429 | 15,985 | 13,727 – 32,786 | 18,414 | 16,156 – 35,215 | 143.2 | 1,085.6 | 953 - 2076 | 0.87 | 0.85 - 0.93 |

[1] 3 week rolling average

[2] Range calculated using average 95% confidence intervals calculated around each VE point estimates as shown in Table S7

[3] Per 100,000 population

[4] Prevented fraction

[5] Symptomatic COVID-19 case presenting to primary care or community testing centre

[6] Emergency Department presentation excluded cases subsequently hospitalised or who were admitted to an intensive care unit or who died

[7] Intensive care unit admission

**Table S9 Averted outcomes in those aged 50 years and older by vaccination status: Week 51 2021 to week 12 2023, Ireland: Sensitivity analysis**

| Outcome[1] | Observed | Averted | Averted range[2] | PC3 | PC range[2] | B1[4] | B1 range[2] | B2[5] | B2 range[2] | B3[6] | B3 range[2] |
|---|---|---|---|---|---|---|---|---|---|---|---|
| Symptomatic case[7] | 86,570 | 48,551 | 27,901 – 70,935 | 3,649 | 2,473 – 5,103 | 42,554 | 148 - 364 | 2,087 | 975 – 3,401 | 260 | 148 - 364 |
| Emergency Department presentation[8] | 8,422 | 9,517 | 6,771 – 12,616 | 634 | 393 - 916 | 7,098 | 237 - 574 | 1,391 | 832 – 1,982 | 394 | 237 - 574 |
| Hospitalisation | 24,578 | 102,160 | 60,594 – 157,142 | 8,117 | 3,773 – 14,092 | 73,362 | 2,805 – 6,642 | 16,268 | 8,922 – 26,152 | 4,414 | 2,805 – 6,642 |
| Intensive Care Unit admission[9] | 392 | 3,303 | 1,782 – 5,222 | 314 | 164 - 508 | 2,150 | 125 - 350 | 611 | 335 - 943 | 228 | 125 - 350 |
| Death | 2,429 | 15,985 | 13,727 – 32,786 | 1,484 | 1,291 – 3,187 | 11,486 | 360 – 1,264 | 2,328 | 1,327 – 4,446 | 688 | 360 – 1,264 |

[1] 3 week rolling average

[2] Range calculated using average 95% confidence intervals calculated around each VE point estimates as shown in Table S7

[3] PC = Outcome averted among those who had primary vaccination course only with range

[4] B1 = Outcome averted among those who had primary vaccination course and booster 1 only with range

[5] B2 = Outcome averted among those who had primary vaccination course, booster 1 and booster 2 only with range

[6] B3 = Outcome averted among those who had primary vaccination course, booster 1, booster 2 and booster 3 only with range

[7] Symptomatic COVID-19 case presenting to primary care or community testing centre

[8] Emergency Department presentation excluded cases subsequently hospitalised or who were admitted to an intensive care unit or who died

[9] Intensive care unit admission

## Epidemiology of COVID-19 in Ireland during study period including whole genome sequencing (WGS) results

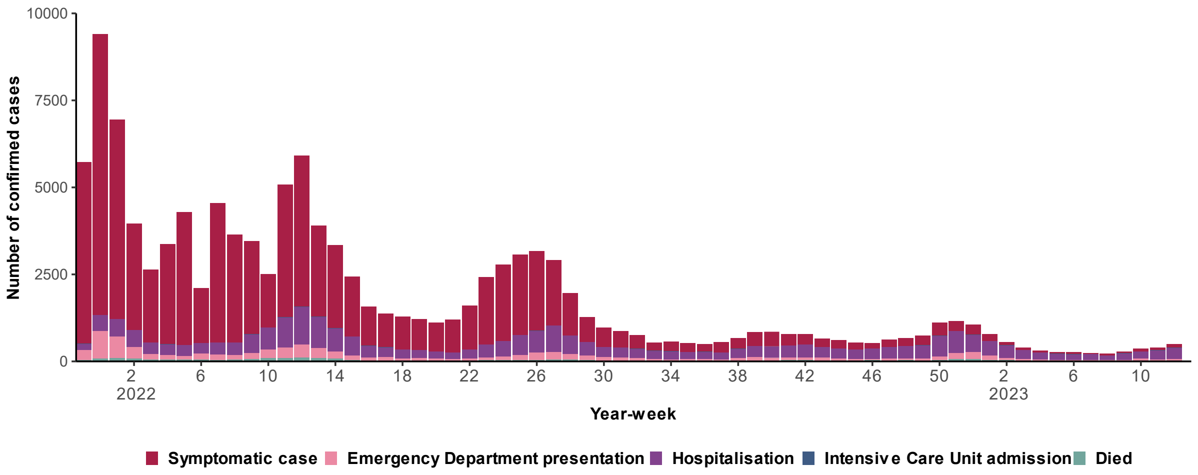


**Figure S2 Confirmed COVID-19 cases by outcome in those aged 50 years and older: Week 51 2021 to week 12 2023, Ireland (n = 121,642)**

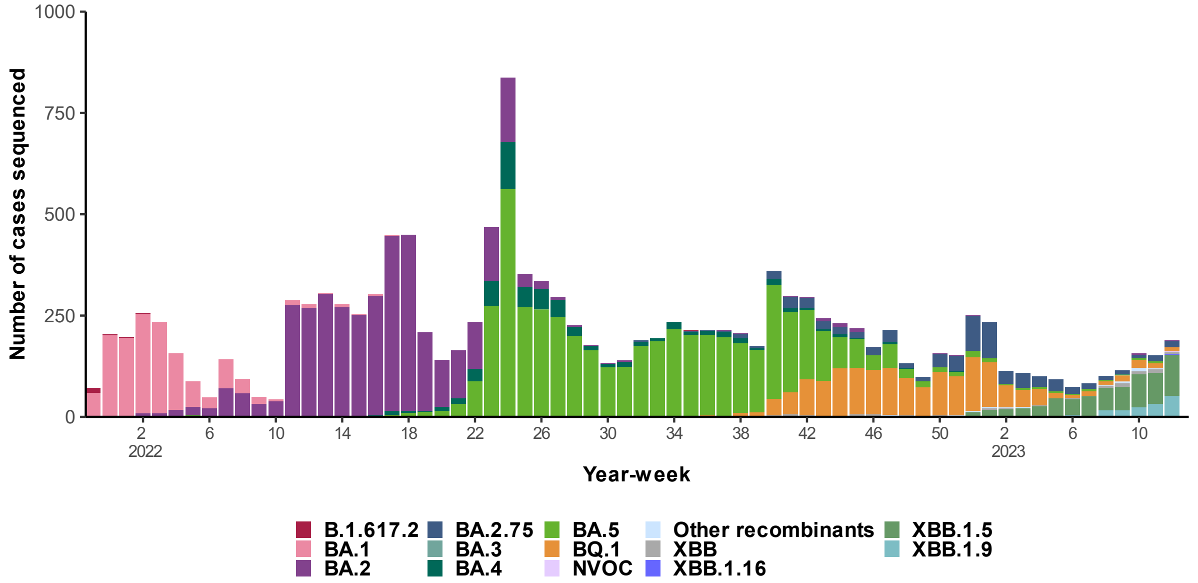


**Figure S3 Confirmed COVID-19 cases by WGS result in those aged 50 years and older who experienced an outcome: Week 51 2021 to week 12 2023, Ireland (n = 13,874)**

## References


1. National Immunisation Advisory Committee. NIAC Immunisation Guidelines: Chapter 5a COVID-19. Ireland: RCPI; 2023.

2. Health Protection Surveillance Centre. COVID-19 Vaccination Uptake in Ireland Weekly Report: Week 24 2023. Ireland: HPSC; 2023.

3. Hafeez MU, Ikram M, Shafiq Z, Sarfraz A, Sarfraz Z, Jaiswal V, et al. COVID-19 Vaccine-Associated Thrombosis With Thrombocytopenia Syndrome (TTS): A Systematic Review and Post Hoc Analysis. Clinical and applied thrombosis/hemostasis : official journal of the International Academy of Clinical and Applied Thrombosis/Hemostasis. 2021;27:10760296211048815. 10.1177/10760296211048815

4. Government of Ireland. National COVID-19 Vaccination Programme: Strategy. Ireland: Government of Ireland; 2020.

5. Health Service Executive National Immunisation Office. Table of recommended groups for COVID-19 autumn booster and flu vaccine. Ireland: HSE; 2023.

6. Department of Health. Strategic Approach for the Management of COVID-19 Preparedness for Autumn/Winter 2022/2023. Ireland: Department of Health; 2022.

7. Health Service Executive. Winter Plan October 2022-March 2023. Ireland: HSE; 2022.

8. International Vaccine Access Centre Johns Hopkins Bloomberg School of Public Health and World Health Organisation and Coalition for Epidemic Preparedness Innovations. Results of COVID-19 Vaccine Effectiveness & Impact Studies: An Ongoing Systematic Review: Methods. Geneva: WHO; 2023.

9. Solante R, Alvarez-Moreno C, Burhan E, Chariyalertsak S, Chiu NC, Chuenkitmongkol S, et al. Expert review of global real-world data on COVID-19 vaccine booster effectiveness and safety during the omicron-dominant phase of the pandemic. Expert Rev Vaccines. 2023;22(1):1-16. 10.1080/14760584.2023.2143347

10. Tamandjou Tchuem CR, Auvigne V, Vaux S, Montagnat C, Paireau J, Monnier Besnard S, et al. Vaccine effectiveness and duration of protection of COVID-19 mRNA vaccines against Delta and Omicron BA.1 symptomatic and severe COVID-19 outcomes in adults aged 50 years and over in France. Vaccine. 2023;41(13):2280-8. 10.1016/j.vaccine.2023.02.062

11. Kirsebom FCM, Andrews N, Sachdeva R, Stowe J, Ramsay M, Lopez Bernal J. Effectiveness of ChAdOx1-S COVID-19 booster vaccination against the Omicron and Delta variants in England. Nature communications. 2022;13(1):7688. 10.1038/s41467-022-35168-7

12. Link-Gelles R, Ciesla AA, Fleming-Dutra KE, Smith ZR, Britton A, Wiegand RE, et al. Effectiveness of Bivalent mRNA Vaccines in Preventing Symptomatic SARS-CoV-2 Infection - Increasing Community Access to Testing Program, United States, September-November 2022. MMWR Morb Mortal Wkly Rep. 2022;71(48):1526-30. 10.15585/mmwr.mm7148e1

13. Thompson MG, Natarajan K, Irving SA, Rowley EA, Griggs EP, Gaglani M, et al. Effectiveness of a Third Dose of mRNA Vaccines Against COVID-19-Associated Emergency Department and Urgent Care Encounters and Hospitalizations Among Adults During Periods of Delta and Omicron Variant Predominance - VISION Network, 10 States, August 2021-January 2022. MMWR Morb Mortal Wkly Rep. 2022;71(4):139-45. 10.15585/mmwr.mm7104e3

14. Ferdinands JM, Rao S, Dixon BE, Mitchell PK, DeSilva MB, Irving SA, et al. Waning 2-Dose and 3-Dose Effectiveness of mRNA Vaccines Against COVID-19-Associated Emergency Department and Urgent Care Encounters and Hospitalizations Among Adults During Periods of Delta and Omicron Variant Predominance - VISION Network, 10 States, August 2021-January 2022. MMWR Morb Mortal Wkly Rep. 2022;71(7):255-63. 10.15585/mmwr.mm7107e2

15. Natarajan K, Prasad N, Dascomb K, Irving SA, Yang DH, Gaglani M, et al. Effectiveness of Homologous and Heterologous COVID-19 Booster Doses Following 1 Ad.26.COV2.S (Janssen [Johnson & Johnson]) Vaccine Dose Against COVID-19-Associated Emergency Department and Urgent Care Encounters and Hospitalizations Among Adults - VISION Network, 10 States, December 2021-March 2022. MMWR Morb Mortal Wkly Rep. 2022;71(13):495-502. 10.15585/mmwr.mm7113e2

16. Tartof SY, Slezak JM, Puzniak L, Hong V, Frankland TB, Xie F, et al. Effectiveness and durability of BNT162b2 vaccine against hospital and emergency department admissions due to SARS-CoV-2 omicron sub-lineages BA.1 and BA.2 in a large health system in the USA: a test-negative, case-control study. The Lancet Respiratory Medicine. 2023;11(2):176-87. 10.1016/S2213-2600(22)00354-X

17. Link-Gelles R, Levy ME, Gaglani M, Irving SA, Stockwell M, Dascomb K, et al. Effectiveness of 2, 3, and 4 COVID-19 mRNA Vaccine Doses Among Immunocompetent Adults During Periods when SARS-CoV-2 Omicron BA.1 and BA.2/BA.2.12.1 Sublineages Predominated - VISION Network, 10 States, December 2021-June 2022. MMWR Morb Mortal Wkly Rep. 2022;71(29):931-9. 10.15585/mmwr.mm7129e1

18. Ferdinands JM, Rao S, Dixon BE, Mitchell PK, DeSilva MB, Irving SA, et al. Waning of vaccine effectiveness against moderate and severe covid-19 among adults in the US from the VISION network: test negative, case-control study. BMJ. 2022;379:e072141. 10.1136/bmj-2022-072141

19. Link-Gelles R, Levy ME, Natarajan K, Reese SE, Naleway AL, Grannis SJ, et al. Estimation of COVID-19 mRNA Vaccine Effectiveness and COVID-19 Illness and Severity by Vaccination Status During Omicron BA.4 and BA.5 Sublineage Periods. JAMA network open. 2023;6(3):e232598-e. 10.1001/jamanetworkopen.2023.2598

20. Tartof SY, Slezak JM, Puzniak L, Hong V, Frankland TB, Ackerson BK, et al. BNT162b2 vaccine effectiveness against SARS-CoV-2 omicron BA.4 and BA.5. The Lancet Infectious Diseases. 2022;22(12):1663-5. 10.1016/S1473-3099(22)00692-2

21. Bozio CH, Butterfield KA, Briggs Hagen M, Grannis S, Drawz P, Hartmann E, et al. Protection From COVID-19 mRNA Vaccination and Prior SARS-CoV-2 Infection Against COVID-19–Associated Encounters in Adults During Delta and Omicron Predominance. The Journal of Infectious Diseases. 2023;227(12):1348-63. 10.1093/infdis/jiad040

22. Baum U, Poukka E, Leino T, Kilpi T, Nohynek H, Palmu AA. High vaccine effectiveness against severe COVID-19 in the elderly in Finland before and after the emergence of Omicron. BMC Infect Dis. 2022;22(1):816. 10.1186/s12879-022-07814-4

23. Carazo S, Skowronski DM, Brisson M, Sauvageau C, Brousseau N, Fafard J, et al. Prior infection- and/or vaccine-induced protection against Omicron BA.1, BA.2 and BA.4/BA.5-related hospitalisations in older adults: a test-negative case-control study in Quebec, Canada. medRxiv : the preprint server for health sciences. 2022:2022.12.21.22283740. 10.1101/2022.12.21.22283740

24. Stowe J, Andrews N, Kirsebom F, Ramsay M, Bernal JL. Effectiveness of COVID-19 vaccines against Omicron and Delta hospitalisation, a test negative case-control study. Nature communications. 2022;13(1):5736. 10.1038/s41467-022-33378-7

25. Sharma A, Oda G, Holodniy M. Effectiveness of Messenger RNA–based Vaccines During the Emergence of the Severe Acute Respiratory Syndrome Coronavirus 2 Omicron Variant. Clinical Infectious Diseases. 2022;75(12):2186-92. 10.1093/cid/ciac325

26. Wan EYF, Mok AHY, Yan VKC, Chan CIY, Wang B, Lai FTT, et al. Effectiveness of BNT162b2 and CoronaVac vaccinations against SARS-CoV-2 omicron infection in people aged 60 years or above: a case–control study. Journal of Travel Medicine. 2022;29(8). 10.1093/jtm/taac119

27. Young-Xu Y, Zwain GM, Izurieta HS, Korves C, Powell EI, Smith J, et al. Effectiveness of mRNA COVID-19 vaccines against Omicron and Delta variants in a matched test-negative case–control study among US veterans. BMJ Open. 2022;12(8):e063935. 10.1136/bmjopen-2022-063935

28. Gram MA, Emborg HD, Schelde AB, Friis NU, Nielsen KF, Moustsen-Helms IR, et al. Vaccine effectiveness against SARS-CoV-2 infection or COVID-19 hospitalization with the Alpha, Delta, or Omicron SARS-CoV-2 variant: A nationwide Danish cohort study. PLoS Med. 2022;19(9):e1003992. 10.1371/journal.pmed.1003992

29. Kislaya I, Machado A, Magalhães S, Rodrigues AP, Franco R, Leite PP, et al. COVID-19 mRNA vaccine effectiveness (second and first booster dose) against hospitalisation and death during Omicron BA.5 circulation: cohort study based on electronic health records, Portugal, May to July 2022. Eurosurveillance. 2022;27(37):2200697. doi:https://doi.org/10.2807/1560-7917.ES.2022.27.37.2200697

30. Yan VKC, Wan EYF, Ye X, Mok AHY, Lai FTT, Chui CSL, et al. Effectiveness of BNT162b2 and CoronaVac vaccinations against mortality and severe complications after SARS-CoV-2 Omicron BA.2 infection: a case–control study. Emerging microbes & infections. 2022;11(1):2304-14. 10.1080/22221751.2022.2114854

31. Park SK, Choe YJ, Jang EJ, Kim RK, Lee SW, Kwon GY, et al. Effectiveness of Heterologous COVID-19 Vaccine Booster in Korean Elderly Population, 2022. Journal of Korean medical science. 2023;38(19):e143. 10.3346/jkms.2023.38.e143

32. Shrotri M, Krutikov M, Palmer T, Giddings R, Azmi B, Subbarao S, et al. Vaccine effectiveness of the first dose of ChAdOx1 nCoV-19 and BNT162b2 against SARS-CoV-2 infection in residents of long-term care facilities in England (VIVALDI): a prospective cohort study. The Lancet Infectious diseases. 2021;21(11):1529-38. 10.1016/s1473-3099(21)00289-9

33. Liu B, Stepien S, Dobbins T, Gidding H, Henry D, Korda R, et al. Effectiveness of COVID-19 Vaccination Against Covid-19 Specific and All-Cause Mortality in Older Australians. Available at SSRN 4445191. 2023.

34. VIEW-Hub. [Available from: https://view-hub.org/resources].
